# Psychometric validation of the Japanese version of the lymphedema functioning, disability, and health questionnaire for upper limb lymphedema: A multicenter cross-sectional study

**DOI:** 10.1097/MD.0000000000049846

**Published:** 2026-07-24

**Authors:** Daigo Sakamoto, Toyohiro Hamaguchi, Rumiko Kato, Kazumasa Miyake, Yasuhide Nakayama, Masahiro Abo

**Affiliations:** a Department of Rehabilitation Medicine, The Jikei University School of Medicine, Tokyo, Japan; b Lymphedema Group, Japanese Association of Supportive Care in Cancer, Tokyo, Japan; c Department of Rehabilitation, Graduate School of Health Science, Saitama Prefectural University, Saitama, Japan; d Division of Rehabilitation Medicine, Shizuoka Cancer Center, Shizuoka, Japan; e Department of Rehabilitation Medicine, Kousei Hospital Lymphedema Treatment Center, Okayama, Japan.

**Keywords:** breast cancer, occupational therapy, quality of life, rehabilitation

## Abstract

Upper limb lymphedema that develops after breast cancer treatment affects not only patients’ physical function and daily activities but also their psychosocial well-being, thereby reducing their health-related quality of life (HRQOL). The Lymphedema Functioning, Disability, and Health Questionnaire for Upper Limb Lymphedema (Lymph-ICF-UL) is a scale that comprehensively evaluates these effects. However, a Japanese version is not yet available. This study aimed to translate and develop a Japanese version of the Lymph-ICF-UL and to verify its psychometric properties in patients with breast cancer-related upper limb lymphedema. The scale was translated in accordance with international guidelines. A total of 208 women from 3 facilities in Japan completed the questionnaire. For test-retest reliability, 142 participants were randomly selected, among whom 131 completed the retest. Content validity was evaluated by 21 experts, and internal consistency and test-retest reliability were verified. For construct validity, exploratory and confirmatory factor analyses were conducted, and convergent and discriminant validities were evaluated using subscales of the 36-Item Short-Form Health Survey Questionnaire. The Japanese version of the Lymph-ICF-UL demonstrated acceptable content validity, high internal consistency, and good test–retest reliability. Exploratory and confirmatory factor analyses provided partial support for the original five-factor structure; however, some model fit indices did not meet conventional thresholds. The forced five-factor model with low-loading items removed showed the lowest Bayesian information criterion. Convergent validity was supported for all predefined hypotheses, whereas discriminant validity was supported for 4 of 9 hypotheses, resulting in confirmation of 64% of all hypotheses. The Japanese version of the Lymph-ICF-UL has been demonstrated to have sufficient reliability and validity. This scale is a practical assessment tool for evaluating the HRQOL in patients with upper limb lymphedema.

## 1. Introduction

Breast cancer is the most common cancer among women, and improved survival due to screening and targeted therapies has led to more patients living with long-term complications.^[1]^ One such complication is breast cancer-related upper limb lymphedema (BCRL), characterized by swelling from excessive tissue fluid accumulation due to impaired lymphatic transport.^[2,3]^ Lymphedema is a chronic, incurable condition that significantly impairs the health-related quality of life (HRQOL), causing physical and psychological burdens.^[4]^ Functional impairments may limit daily activities, employment, and leisure. Furthermore, changes in the appearance of the affected limb can lower self-esteem, discourage going out, and restrict social interaction.^[5]^

To maintain and improve HRQOL in patients with BCRL, healthcare providers must accurately assess the difficulties and social constraints patients experience daily and implement appropriate lymphedema management. Although clinical settings commonly use methods such as circumference measurements of the affected limb using a measuring tape and volume measurements, these approaches do not fully capture the perceived health impairments or social restrictions experienced by patients.^[6]^ Patient-reported outcomes (PROs) help to understand changes in HRQOL resulting from physical function improvements.^[7]^ Healthcare has shifted toward a patient-centered model that prioritizes self-reported experiences.^[8]^ PRO-based HRQOL evaluations measure treatment outcomes and amplify patients’ voices, making them essential for evidence-based care.

For HRQOL assessment, scales that accurately capture lymphedema-specific symptoms are essential. Representative HRQOL assessment tools include the Medical Outcomes Study 36-Item Short-Form Health Survey (SF-36), the European Organization for Research and Treatment of Cancer Quality of Life Questionnaire-Core 30, and the Functional Assessment of Cancer Therapy-Breast. These are widely used in clinical practice as PROs with high utility.^[9,10]^ However, they lack sensitivity to BCRL-specific issues, underscoring the need for symptom-specific tools.^[11]^ Conversely, the Lymphedema Functioning, Disability, and Health Questionnaire for Upper Limb Lymphedema (Lymph-ICF-UL) is an HRQOL assessment scale that quantifies functional impairments, activity limitations, and participation restrictions associated with BCRL.^[12]^ This scale is based on the framework of the International Classification of Functioning, Disability, and Health (ICF) developed by the World Health Organization. The Lymph-ICF-UL has been identified as a tool capable of comprehensively and accurately assessing HRQOL in patients with BCRL.^[13,14]^ Originally developed in Dutch, the scale has been translated and adapted for use in several countries to facilitate international clinical application.^[15–18]^ Currently, a Japanese version has not yet been developed. This study aimed to translate and validate a Japanese version of the Lymph-ICF-UL and assess its psychometric properties. Based on the ICF framework, this version will offer a comprehensive tool for evaluating functional impairments, activity limitations, and participation restrictions in patients with BCRL, thereby enabling reliable HRQOL assessment in clinical and research settings for patients with upper limb lymphedema.

## 2. Methods

### 2.1. Study design

This study employed a multicenter cross-sectional survey design in collaboration with 3 Japanese institutions: Tokyo Jikei University Hospital, Shizuoka Cancer Center, and Kousei Hospital Lymphedema Treatment Center.

### 2.2. Ethical considerations

All patients provided written informed consent to participate. The study was approved by the Ethics Committee of Jikei University School of Medicine (approval number: 34-388-11545).

### 2.3. Procedures

#### 2.3.1. Procedure 1: translation of lymph-ICF-UL

The translation followed the guidelines of the International Society for Pharmacoeconomics and Outcomes Research.^[19]^ After obtaining permission from the original developers, the scale and manual underwent forward translation, reconciliation, back-translation, and review by the original developers to ensure semantic equivalence. The same process was applied to the scale and its manual.

Cognitive debriefing was conducted using the developer-approved prototype. Ten patients with BCRL who provided written consent participated. Reading and response times were recorded, and participants rated 4 aspects: clarity of instructions/options, scale comprehensiveness, relevance, and representation of health status on an 11-point scale (0 = not at all agree to 10 = strongly agree). Mean scores ≥7.0 were considered acceptable. They also provided free-text feedback. Patients’ age and sex were recorded. Based on these results, the principal investigator revised the scale, which was then reverse-translated and reviewed by the developers. The Japanese version of the Lymph-ICF-UL was subsequently finalized.

### 2.4. Procedure 2: psychometric validation

#### 2.4.1. Setting

The questionnaire survey and clinical evaluation were conducted by 12 occupational therapists: 2 from Tokyo Jikei University Hospital, 6 from Shizuoka Cancer Center, and 4 from Kousei Hospital Lymphedema Treatment Center. The mean (± standard deviation [SD]) of their clinical experience was 15.3 ± 9.3 years. Each facility had at least 2 therapists who had completed the Ministry of Health, Labor and Welfare-approved education and training program on lymphedema or held certification as lymphedema insurance practitioners from the Japanese Lymphedema Society. The remaining therapists received adequate guidance from these certified professionals and had sufficient knowledge and clinical skills related to lymphedema. Questionnaire surveys, clinical evaluations, and collection of patient medical information were conducted from April 1, 2023, to November 30, 2024.

#### 2.4.2. Participants

Eligibility criteria included a diagnosis of secondary upper limb lymphedema associated with breast cancer, age ≥18 years, and receipt of treatment at one of the study facilities. Exclusion criteria included missing data, cognitive impairment, severe psychological disorders, or systemic conditions that made evaluation difficult. Patients who met the criteria and provided informed consent were enrolled in the study. The recommended sample size for questionnaire development and validation is at least 5 times the number of items, with a minimum of 100 participants.^[20,21]^ The Lymph-ICF-UL contains 29 items; therefore, a sample size of 203 was calculated based on a recommendation of 7 participants per item to ensure excellent validation.

#### 2.4.3. Questionnaire

The questionnaire survey utilized the Japanese versions of the Lymph-ICF-UL and SF-36. The Lymph-ICF-UL is a 29-item scale assessing HRQOL in patients with BCRL, covering 5 domains: physical function, mental function, household activities, mobility activities, and life and social activities. Each item is rated on an 11-point numeric rating scale reflecting the experience of the respondent over the past 2 weeks, with scores converted to a 100-point scale, where lower scores indicate better HRQOL.^[22]^ The SF-36 is a comprehensive HRQOL scale across 8 subcategories, rated on 3- to 5-point Likert scales. Scores range up to 100, with higher scores indicating better health.^[10]^

In the first survey, all participants completed the Lymph-ICF-UL and SF-36 questionnaires at the research facility. In the second survey, participants were randomly selected using a random number table to complete the Lymph-ICF-UL again at home. This follow-up survey occurred 1 week after the initial survey. Participants also evaluated changes in their health status using a five-point anchor question (1 = very good, 2 = somewhat better, 3 = no change, 4 = slightly worse, and 5 = much worse). Completed questionnaires were returned by mail.

#### 2.4.4. Participant characteristics

Basic information collected included sex, age, body mass index, dominant hand, highest level of education, employment status, and whether participants had a spouse or children. The Barthel index was used to assess activities of daily living, with scores ranging from 0 (complete dependence) to 100 (complete independence).^[23]^ Medical data included the patient’s breast cancer status, whether they had undergone axillary lymph node dissection, breast reconstruction surgery, and their treatment history (chemotherapy, radiation therapy, targeted therapy, and hormone therapy). For lymphedema, we recorded the affected side, duration since onset, and classification according to the International Society of Lymphology.

#### 2.4.5. Assessing the content validity

Content validity was evaluated following the Consensus-based Standards for the Selection of Health Measurement Instruments manual.^[24]^ Twenty-one experts involved in the treatment of BCRL participated, including 1 breast surgeon, 1 radiologist, 1 vascular surgeon, 1 plastic surgeon, 1 medical oncologist, 4 rehabilitation physicians, 3 nurses, 4 physical therapists, 4 occupational therapists, and 1 masseur. These experts were not involved in the translation process. The Japanese version of the Lymph-ICF-UL questionnaire was distributed online to experts who provided informed consent. Relevance was assessed using 3 items covering construct, population, and context appropriateness, whereas comprehensiveness was evaluated with 1 item assessing inclusion of all key concepts. Responses were rated on a 4-point Likert scale (1 = very inappropriate, 2 = inappropriate, 3 = appropriate, and 4 = very appropriate). Responses were collected for each domain and the overall scale.

#### 2.4.6. Statistical analysis

Descriptive statistics were used for clinical evaluations and medical information. The Shapiro–Wilk test was used to assess the normality of data distributions. Depending on the distribution, data are expressed as mean and SD or median and interquartile range.

Content validity was assessed by 21 experts using the item-level content validity index (I-CVI) and scale-level content validity index (S-CVI). I-CVI was calculated as the proportion of experts rating an item as “3: Quite relevant” or “4: Highly relevant.” S-CVI was calculated as the average of I-CVIs (S-CVI/Average) and as the proportion of items with I-CVI ≥ 0.78 (S-CVI/Universal Agreement). Content validity was considered high when I-CVI ≥ 0.78 and S-CVI ≥ 0.80.^[25]^

Internal consistency was evaluated using Cronbach’s α for each domain and the total score. Values ≥0.7 were considered “acceptable” and ≥0.8 “good.”^[26]^ Test-retest reliability was evaluated using the intraclass correlation coefficient (ICC) between 2 measurements, with ICC ≥0.75 indicating “good” reliability.^[27]^ Descriptive statistics were used to examine changes in health status during the retest. Measurement error was calculated using the standard error of measurement (SEM), with values <10% of the score range considered acceptable.^[28]^


$$SEM=SD\times \sqrt{1-ICC}$$

Construct validity was evaluated using exploratory factor analysis (EFA) and confirmatory factor analysis (CFA). EFA employed free and forced extraction based on the original five-factor structure, using maximum likelihood extraction and Promax rotation. The number of factors was determined by the Kaiser-Meyer-Olkin (KMO) index and Bartlett’s test of sphericity; KMO ≥0.7 was considered appropriate. Model fit was assessed using the Tucker-Lewis Index (TLI), Root Mean Square Error of Approximation (RMSEA), and Bayesian Information Criterion (BIC), with TLI ≥0.90, RMSEA ≤ 0.08, and lower BIC indicating better fit.^[29,30]^ Factor purity was assessed by checking for cross-loadings (loadings ≥0.40 on multiple factors).^[26]^ Items with loadings < 0.40 were retained if theoretically appropriate, particularly those between 0.35 and 0.39 without cross-loading. Items with loadings <0.35 were reviewed by 3 institutional representatives and retained if deemed important to HRQOL in BCRL. Based on EFA results, CFA was conducted to confirm factor structure, comparing the EFA-derived model with the original five-factor model. For items with loadings <0.35 in EFA, CFA compared models with and without these items. CFA used the maximum likelihood method and assessed model fit using the Comparative Fit Index (CFI), TLI, RMSEA, Standardized Root Mean Square Residual (SRMR), and BIC. CFI and TLI ≥0.90 and RMSEA and SRMR ≤0.08 were considered acceptable.^[31,32]^

Convergent and discriminant validity were assessed by correlation analysis between the Lymph-ICF-UL domain scores and SF-36 scores based on hypotheses from the original Lymph-ICF-UL development study.^[12]^ The strength of the correlation was interpreted based on the absolute value of the correlation coefficient as follows: < 0.4 = weak, 0.4–0.74 = moderate, 0.75–0.9 = strong, and ≥0.9 = very strong.^[33]^ Convergent validity was supported when corresponding domains showed moderate-to-very strong correlations; discriminant validity was supported when the unrelated domains showed weak correlations. Validity was rated “very good” if ≥90% of 14 hypotheses were confirmed, “good” if 75% to 89%, and “moderate” if 40% to74%.^[22]^ All analyses were conducted using Jamovi version 2.2.1 (https://www.jamovi.org). Statistical significance was set at *P *< .05.

## 3. Results

### 3.1. procedure 1: translation of lymph-ICF-UL

The scale was translated following standard procedures with cultural adaptation in mind. During this process, certain expressions were adjusted. The original developers noted that the translation of item 4 (“Do you have numbness?”) differed from the original. The forward translators revised the item through discussion, and after back-translation, the developers approved the revision.

A pretest was conducted with 10 women with BCRL (median age: 61.5 [54.3, 66.8]) from 3 research sites. Mean (± SD) times were 72.9 ± 27.8 seconds to read the instructions, 207 ± 58.1 seconds to respond, and 280 ± 75.7 seconds in total. Participants rated clarity (8.6 ± 1.2), comprehensiveness (8.5 ± 1.3), relevance (7.8 ± 1.9), and representation of their health status (8.9 ± 1.5), all exceeding the threshold of 7.0. Participants provided several comments. For instance, the circles for “not applicable” were considered too large and were resized to reduce the burden. Some participants felt that the 11-point Numeric rating scale had too many options; however, it was retained for consistency with the original scale. Comments on terminology were discussed, and the terms were deemed appropriate without modification. These revisions confirmed the scale’s content and cross-cultural validity, leading to the completion of the Japanese version of the Lymph-ICF-UL (Supplemental Digital Content 1, Supplemental Digital Content 1).

### 3.2. Procedure 2: psychometric validation

#### 3.2.1. Participants

**Figure 1.**
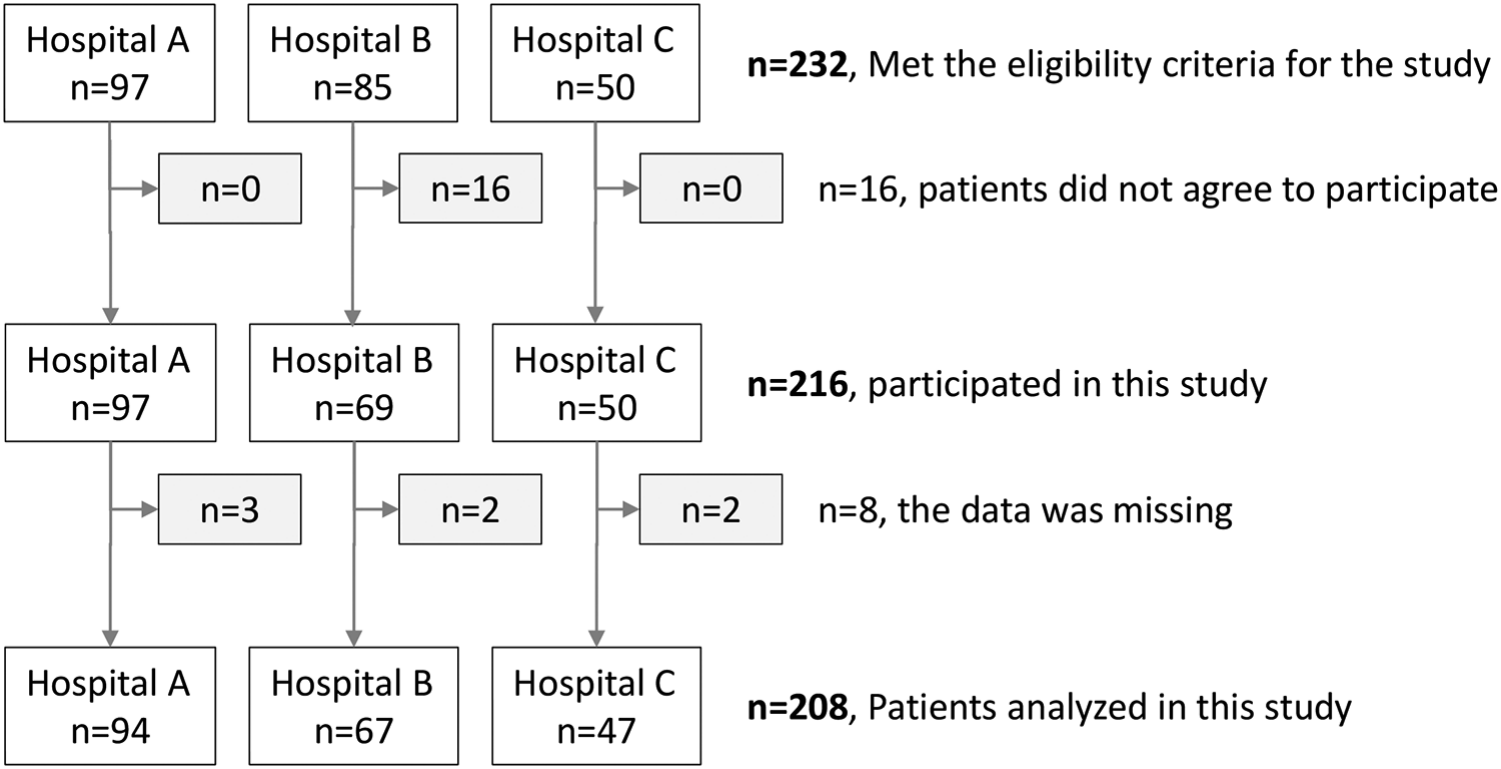
Patient selection procedure.

A total of 232 individuals were invited to participate in the study across 3 facilities, and consent was obtained from 216 participants. Eight participants with missing data were excluded, resulting in a final sample of 208 participants (Fig. 1). All participants were female, with a mean age of 60.81 years and a median body mass index of 22.7 kg/m^2^. Right-handedness was predominant, and the median Barthel Index score was 100. The medical information included breast cancer stage, primarily stages II and III, with most participants having undergone axillary lymph node dissection. According to the International Society of Lymphology, lymphedema staging was predominantly early stage 2 and late stage 2, with a median duration since onset of 24 months. Detailed clinical characteristics of the participants are presented in Table 1.

**Table 1:** Clinical characteristics of the analyzed patients.

Characteristics		All (n = 208)
Sex	Female:Male	208 (100):0 (0)
Age (yr)		60.8 ± 10.9
Body mass index (kg/m^2^)		22.7 (20.6, 25.8)
Dominant hand	Left:Right	11 (5.3):197 (94.7)
Education level	Middle school	5 (2.4)
	High school	81 (38.9)
	Technical school	51 (24.5)
	University	67 (32.2)
	Graduate school	4 (1.9)
Employment status	Employed	119 (57.2)
	Unemployed	89 (42.8)
Marital status	Married	166 (79.8)
	Single/divorced	42 (20.2)
Presence or absence of children	Yes	128 (61.5)
	No	80 (38.5)
Barthel index		100 (100)
Cancer staging	0	1 (0.5)
	I	5 (2.4)
	II	103 (49.5)
	III	94 (45.2)
	IV	5 (2.4)
Surgical side	Left:Right:Bilateral	111 (53.4):94 (45.2):3 (1.4)
Post-operative period (mo)		68 (35, 114)
Axillary lymph node dissection	Yes	198 (95.2)
	No	10 (4.8)
Breast reconstruction	Yes	17 (8.2)
	No	191 (91.8)
Neoadjuvant chemotherapy	Yes	73 (35.1)
	No	135 (35.1)
Treatments received after surgery		
Chemotherapy	Yes	136 (65.4)
	No	72 (34.6)
Radiation therapy	Yes	153 (73.6)
	No	55 (26.4)
Targeted therapy	Yes	20 (9.6)
	No	188 (90.4)
Endocrine therapy	Yes	117 (56.3)
	No	91 (43.8)
ISL staging of lymphedema	Stage 1	7 (3.4)
	Early-stage 2	135 (64.9)
	Late-stage 2	66 (31.7)
	Stage 3	0 (0)
Affected side	Left:Right:Bilateral	111 (53.4):94 (45.2):3 (1.4)
Duration since onset of lymphedema (mo)		24 (6, 55)

Values are presented as n (%) or mean ± standard deviation or median (25th, 75th percentile).

ISL = international society of lymphology.

Regarding content validity, responses were obtained from all experts who were invited to participate in the survey. A total of 21 experts responded, with a mean (± SD) of 23.4 ± 12.2 years of experience in clinical and research work. The I-CVI results showed that, except for Question 1 (household activities) and Question 3 (overall), all items had I-CVI scores of ≥0.78. The S-CVI/Average and S-CVI/Universal Agreement were ≥0.80 for all items (Table 2).

**Table 2:** Item-level content validity index of the Lymph-ICF-UL.

Index		Question 1	Question 2	Question 3	Question 4
I-CVI	Physical functions	0.81	0.76	0.81	0.71
	Mental functions	0.91	0.86	0.91	0.86
	Household activities	0.71	0.81	0.86	0.81
	Mobility activities	0.91	0.86	0.91	0.91
	Life and social activities	0.95	0.95	0.95	0.91
	Overall	0.91	0.91	0.71	0.91
S-CVI/Average		0.86	0.85	0.89	0.84
S-CVI/Universal Agreement		0.80	0.80	1.00	0.80

Questionnaire items:

Question 1: “Did you find the explanations and choices in the questionnaire easy to understand? Question 2: “Did the questionnaire include information about your physical and mental condition, daily activities, household chores, and social life? Question 3: “Was the content of the questionnaire relevant to your health condition and past experiences? Question 4: “Was the questionnaire able to accurately reflect your health status?

I-CVI = item-level content validity index, Lymph-ICF-UL = lymphedema functioning, disability, and health questionnaire for upper limb lymphedema, SCV-I = scale-level content validity index.

#### 3.2.2. Reliability and validity

Cronbach’s α was calculated to assess internal consistency, and all domains, as well as the overall scale, showed values of ≥0.8 (Table 3). To evaluate test–retest reliability, 142 participants were asked to complete the questionnaire a second time, and 131 responses were received, resulting in a response rate of 92.3%. The mean (± SD) score for the anchor question regarding changes in health status between the 2 measurements was 3.40 ± 0.54. The ICC values for all domains and the overall scale were ≥0.75, indicating good test–retest reliability. SEM was calculated to assess measurement error, and for all 4 domains and the overall scale, except for the mental functions domain, SEM values were <10% of the score range (Table 3).

**Table 3:** Reliability of the total score and domain scores of the Lymph-ICF-UL.

Score	Cronbach’s α	ICC (95% CI)	SEM
Physical functions	0.906	0.894 (0.861, 0.920)	7.500
Mental functions	0.868	0.827 (0.775, 0.868)	11.786
Household activities	0.893	0.795 (0.735, 0.842)	8.735
Mobility activities	0.889	0.902 (0.871, 0.925)	6.561
Life and social activities	0.805	0.849 (0.803, 0.885)	7.813
Total	0.955	0.931 (0.908, 0.948)	4.802

CI = confidence interval, ICC = intraclass correlation coefficient, Lymph-ICF-UL = lymphedema functioning, disability, and health questionnaire for upper limb lymphedema, SEM = standard error of measurement.

An EFA was conducted to verify construct validity. In the exploratory-derived model and the forced five-factor model, the KMO index was ≥0.7, and Bartlett’s sphericity test yielded a *P*-value of <.01, confirming that the data were suitable for factor analysis. RMSEA, TLI, and BIC were calculated as indicators of model fit. The results showed that RMSEA exceeded 0.08 for both models, and TLI was <0.90. Conversely, the BIC was smaller for the forced five-factor model, indicating a relatively better fit (Table 4). In the cross-loading analysis, the factor loading of item 18 in the exploratory-derived model was <0.40 (but >0.35); however, it was retained based on theoretical validity. All other items were loaded clearly onto a single factor. In the forced five-factor model, item 29 was loaded onto multiple factors but was assigned to the factor with the highest loading. Item 11 had a low factor loading of 0.22 but was retained because it was considered theoretically important for assessing stress. Details of factor loadings are presented in Supplemental Digital Content 2, Supplemental Digital Content 2. Subsequently, CFA was conducted based on the EFA results. In the CFA, 3 models were compared: a model based on the forced five-factor model with low-loading items deleted, a model retaining those items, and a model replicating the original five-factor structure. All models passed the model fit test (*P* < .01), confirming their suitability for factor analysis. The fit indices showed that CFI and TLI were < .90 for all models, SRMR was ≤0.08, and RMSEA was >0.08. Among the models, the BIC indicated that the forced five-factor model with low-loading factor items removed demonstrated the best fit (Table 5). Furthermore, to verify convergent and discriminant validity, a correlation analysis was conducted between the Lymph-ICF-UL and SF-36 scores. These data were not normally distributed; therefore, Spearman’s rank correlation coefficients were used. Convergent validity was supported for all 5 hypotheses (100%), and discriminant validity was supported for 4 out of 9 hypotheses (44%) (Table 6, Supplemental Digital Content 3, Supplemental Digital Content 3). These results confirmed 64% of the overall hypotheses, indicating that the convergent and discriminant validities were moderate.

**Table 4:** Model fit statistics for exploratory factor analysis.

Model	KMO	Bartlett’s Test			RMSEA (95% CI)	TLI	BIC
		*X* ^2^	df	*P*			
Exploratory-derived model	0.837	2481	406	<.01	0.129 (0.119, 0.143)	0.709	–595
Forced five-factor model	0.837	2481	406	<.01	0.114 (0.102, 0.129)	0.770	–629

BIC = Bayesian information criterion, CI = confidence interval, KMO = Kaiser-Meyer-Olkin, RMSEA = root mean square error of approximation, TLI = Tucker-Lewis index.

**Table 5:** Model fit statistics for confirmatory factor analysis.

Model		Model fit			CFI	TLI	SRMR	RMSEA (95% CI)	BIC
		*X* ^2^	df	*P*					
EFA model with forced five-factor extraction	Full-item model	973	367	<.01	0.849	0.833	0.0710	0.0891 (0.0823, 0.0959)	24,224
	Item-deleted model	947	340	<.01	0.848	0.831	0.0718	0.0926 (0.0857, 0.0997)	23,454
Original five-factor structure model		1038	367	<.01	0.833	0.815	0.0774	0.0937 (0.0870, 0,1000)	24,289

BIC = Bayesian information criterion, CFI = comparative fit index; CI = confidence interval, EFA = exploratory factor analysis, RMSEA = root mean square error of approximation, SRMR = standardized root mean square residual; TLI = Tucker-Lewis index.

**Table 6:** Correlations between Lymph-ICF-UL domain scores and SF-36 subscale scores for assessing convergent and discriminant validity.

Lymph-ICF-UL domain	SF-36 subscale	Hypothesis type	Spearman’s ρ	*P*
Physical functions	Bodily pain	Convergent	–0.514*	<.001
Physical functions	Role-emotional	Discriminant	–0.371*	<.001
Physical functions	Mental health	Discriminant	–0.332*	<.001
Mental functions	Physical functioning	Discriminant	–0.278*	<.001
Mental functions	Role-physical	Discriminant	–0.318*	<.001
Mental functions	Mental health	Convergent	–0.476*	<.001
Household activities	Physical functioning	Convergent	–0.512*	<.001
Household activities	Role-emotional	Discriminant	–0.457	<.001
Household activities	Mental health	Discriminant	–0.421	<.001
Mobility activities	Physical functioning	Convergent	–0.638*	<.001
Mobility activities	Role-emotional	Discriminant	–0.486	<.001
Mobility activities	Mental health	Discriminant	–0.418	<.001
Life and social activities	Physical functioning	Discriminant	–0.612	<.001
Life and social activities	Social functioning	Convergent	–0.552 †	<.001

* indicates that the correlation met the prespecified hypothesis. The hypotheses were derived from the original Lymph-ICF study and operationalized for the revised NRS-based Lymph-ICF-UL.^[12,22]^ The strength of the correlation was interpreted based on the absolute value of the correlation coefficient as follows: <0.4 = weak, 0.4–0.74 = moderate, 0.75–0.9 = strong, and ≥0.9 = very strong. Convergent validity was supported when corresponding domains showed moderate-to-very strong correlations; discriminant validity was supported when the unrelated domains were weakly correlated.

Lymph-ICF-UL = lymphedema functioning, disability, and health questionnaire for upper limb lymphedema; SF-36 = 36-item short-form health survey questionnaire.

## 4. Discussion

We developed a Japanese version of the Lymph-ICF-UL to evaluate the HRQOL of patients with BCRL and verified its reliability and validity. Good results were obtained for content validity, internal consistency, and test-retest reliability. Regarding construct validity, this version demonstrated a similar five-factor structure to the original version, with good convergent and moderate discriminant validity. These results indicate that this version of the Lymph-ICF-UL is a useful tool for assessing HRQOL in patients with BCRL in clinical and research settings in Japan. Given the growing emphasis on patient-centered care and PROs, a psychometrically evaluated Japanese version may enable standardized assessment of BCRL-related functioning and participation, thereby supporting clinical practice and multicenter research in Japan.

In addition, we developed this Japanese version of the Lymph-ICF-UL to ensure translation validity and cultural appropriateness, using a method based on the International Society for Pharmacoeconomics and Outcomes Research guidelines. The responses obtained from the cognitive debriefing generally met the benchmark of ≥7.0, and terms that raised questions from respondents were reviewed by the translators and confirmed to be appropriately translated. The median total time required for participants to complete the questionnaire was 280 seconds, supporting the manual’s stated duration of approximately 5 minutes. In conclusion, the Japanese version of the Lymph-ICF-UL is an easily understandable assessment scale for patients with BCRL and can be appropriately used in clinical settings, having been developed while maintaining cross-cultural validity.

In the content validity verification, 21 experts with an average practical experience of 23.4 years participated. The I-CVI results showed that most items scored ≥0.78, meeting the criteria generally considered acceptable for validity. Furthermore, S-CVI/Average and S-CVI/Universal Agreement were ≥0.80, indicating that the scale as a whole demonstrated good content validity. However, 2 items with I-CVI scores <0.78 may have characteristics prone to individual differences in evaluation. Particularly, in the “household activities” category, the specific content of household activities varies widely depending on culture and lifestyle, and it is speculated that cultural differences between Japan and the West possibly contributed to evaluation variability.^[34,35]^ However, other evaluation indicators showed high values, and no response difficulties were identified in the cognitive debriefing. Therefore, the original structure was retained. In conclusion, the Japanese version of the Lymph-ICF-UL has content validity for comprehensively assessing the HRQOL of patients with BCRL, with cultural considerations appropriately accounted for.

In the evaluation of internal consistency, the Cronbach’s α coefficient for the entire scale was 0.958, and the domains showed good values ranging from 0.805 to 0.906. These results indicate that each domain consistently measures the same concept, suggesting that the internal consistency of the scale is adequately ensured. These findings are consistent with those reported for the original Lymph-ICF-UL and other translated versions, which also demonstrated excellent internal consistency across domains.^[12,15–17]^ Generally, when the α coefficient is >0.95, redundancy among items is suspected; however, the α coefficients of the domains were <0.95, and since each item is constructed to focus on different aspects, it is inferred that content overlap is limited.

In the evaluation of retest reliability, the overall ICC for the scale was 0.931, and the ICCs for the domains ranged from 0.795 to 0.902, all of which were >0.75, indicating good reliability. The response rate at retest was high at 92.3%, and the results of the anchor questions regarding changes in health status (3.40 ± 0.54) fell within the range corresponding to “no change,” indicating that retesting was conducted under stable conditions. The SEM was <10% of the score range for all domains and the overall scale, except for mental functions, indicating that the error was within a practically acceptable range. The relatively high SEM observed in mental functions suggests that the measurement of psychological factors may be more susceptible to individual mental states.^[36]^ These results indicate that the Japanese version of the Lymph-ICF-UL is highly reliable in terms of reproducibility and measurement accuracy and is suitable for repeated measurements. Similar levels of test-retest reliability have been reported in previous validation studies of the original and translated versions of the Lymph-ICF-UL, supporting the stability of the instrument across different cultural settings.^[12,15–17]^

Regarding structural validity, a five-factor structure was evaluated using EFA and CFA. In the EFA, an exploratory-derived model based on free extraction and a forced five-factor model based on the original version were compared, and the latter showed a relatively better fit in terms of BIC. Furthermore, many items were loaded clearly onto a single factor, and items with cross-loadings or low factor loadings were deemed appropriate based on content validity and theoretical relevance. Therefore, the EFA results support the notion that the Japanese version of the scale has a consistent set of constructs. In the CFA, 3 models (forced five-factor, model with low factor loadings removed, and original five-factor) were compared. All models had CFI and TLI < 0.90 and RMSEA > 0.08; however, SRMR was < 0.08 for all models, and BIC was the smallest for the model with items with low factor loadings removed. Although CFI, TLI, and RMSEA did not meet conventional thresholds, SRMR was acceptable, and BIC supported the forced five-factor model after excluding low-loading items. Considering theoretical consistency with the original instrument and cross-cultural comparability, we retained the original five-factor structure while acknowledging the need for further validation. Items with low factor loadings were consistent with the theoretical background of their respective factors, and further consideration is required regarding the handling of these items. These results indicate that the five-factor structure, which is consistent with the original version, is effective in maintaining the theoretical framework and content validity of the Japanese version of the Lymph-ICF-UL and that construct validity is supported by statistical indicators and theoretical validity. The retention of the original five-factor structure is consistent with the conceptual framework proposed by the original developers and supports the cross-cultural applicability of the instrument.^[12,15]^

Regarding convergent validity, all hypotheses were supported, indicating that the Lymph-ICF-UL and SF-36 accurately measured the common constructs. This finding is consistent with the results of previous validation studies of disease-specific HRQOL instruments for lymphedema, which have demonstrated strong associations with generic quality-of-life measures.^[12,15,17]^ Conversely, only 44% of the hypotheses were supported in terms of discriminant validity, which is attributed to the lack of clear factor independence between Lymph-ICF-UL’s “Mental functions” and SF-36’s “Vitality” and “Emotional role function.” These results suggest that BCRL reflects a disease-specific characteristic where physical impairments are strongly associated with psychological aspects. In particular, the findings highlight the multidimensional nature of BCRL, in which physical symptoms, activity limitations, and psychological well-being are closely interconnected.^[37,38]^ Therefore, complete independence between physical and mental health domains may not be expected when assessing HRQOL in patients with BCRL.

## 5. Clinical implications

The Japanese version of the Lymph-ICF-UL is a reliable and valid outcome measure for evaluating patients with BCRL and measuring treatment efficacy. This allows healthcare professionals to comprehensively and accurately assess the HRQOL of patients, enabling the provision of rehabilitation treatments tailored to individual needs. Furthermore, this scale contributes to the promotion of interventions and international comparative research. To advance clinical implementation, it is essential to validate the development of a shorter version and the transition to an electronic response format, with particular emphasis on ease of use and reduction of burden for elderly patients and those with complex sociopsychological backgrounds.^[39]^

## 6. Study limitations

This study has some limitations worth noting. First, it was limited to 3 facilities in Japan and did not include severe cases; therefore, additional verification is required for generalization to other regions or different severity levels. Second, in the verification of structural validity, some fit indices did not meet the criteria, and items with low factor loadings and cross-loadings were identified. To improve item composition and model structure, further validation with a larger sample and a more detailed examination of item characteristics are required. Third, in the convergent and discriminant validity verification, some hypotheses were not supported, suggesting a conceptual overlap between scales and the influence of lymphedema-specific physical and psychological factors. Fourth, this study used a cross-sectional design, and its responsiveness and predictive validity have not been verified. To enhance the practicality of the Japanese version of the Lymph-ICF-UL, it is necessary to verify the association between score changes induced by intervention and clinical outcomes.

## 7. Conclusions

We translated and developed the Japanese version of the Lymph-ICF-UL, confirming high levels of content validity, internal consistency, and test-retest reliability and obtaining some support for construct validity. This scale is a useful tool for comprehensively evaluating the HRQOL of patients with BCRL. Based on the ICF framework, it complements traditional physical function assessments and applies to clinical evaluations and research outcomes.

## Acknowledgments

We thank the occupational therapists at Jikei University Hospital, Shizuoka Cancer Center, and Kousei Hospital Lymphedema Treatment Center for their support with data collection. We sincerely thank the experts from the Japanese Association of Rehabilitation Medicine, Japanese Association of Supportive Care in Cancer, Japanese Lymphedema Society, and Japanese Society for Lymphedema Therapy for their valuable input in evaluating the scale’s content validity.

## Author contributions

**Conceptualization:** Daigo Sakamoto, Toyohiro Hamaguchi, Masahiro Abo.

**Data curation:** Daigo Sakamoto, Rumiko Kato, Kazumasa Miyake, Yasuhide Nakayama.

**Formal analysis:** Daigo Sakamoto, Kazumasa Miyake, Toyohiro Hamaguchi.

**Funding acquisition:** Daigo Sakamoto.

**Investigation:** Daigo Sakamoto, Rumiko Kato, Yasuhide Nakayama.

**Methodology:** Daigo Sakamoto, Toyohiro Hamaguchi, Masahiro Abo.

**Resources:** Daigo Sakamoto, Rumiko Kato, Kazumasa Miyake.

**Visualization:** Daigo Sakamoto.

**Validation:** Rumiko Kato, Kazumasa Miyake, Toyohiro Hamaguchi.

**Supervision:** Yasuhide Nakayama, Toyohiro Hamaguchi, Masahiro Abo.

**Project administration:** Masahiro Abo.

**Writing – original draft:** Daigo Sakamoto.

**Writing – review & editing:** Kazumasa Miyake, Yasuhide Nakayama, Toyohiro Hamaguchi, Masahiro Abo.







## References

[1] Giaquinto AN Sung H Newman LA. Breast cancer statistics 2024. CA Cancer J Clin. 2024;74:477–95. 39352042 10.3322/caac.21863

[2] Manrique OJ Bustos SS Ciudad P. Overview of lymphedema for physicians and other clinicians: a review of fundamental concepts. Mayo Clin Proc. 2022;97:1920–35. 32829905 10.1016/j.mayocp.2020.01.006

[3] Jeong SH Chun SM Kim M. Multimodal treatments and the risk of breast cancer-related lymphedema: insights from a nationally representative cohort in South Korea. BMC Cancer. 2025;25:114. 39844110 10.1186/s12885-025-13513-5 PMC11753110

[4] Jørgensen MG Toyserkani NM Hansen FG Bygum A Sørensen JA. The impact of lymphedema on health-related quality of life up to 10 years after breast cancer treatment. NPJ Breast Cancer. 2021;7:70. 34075045 10.1038/s41523-021-00276-y PMC8169644

[5] Anbari AB Wanchai A Armer JM. Breast cancer-related lymphedema and quality of life: a qualitative analysis over years of survivorship. Chronic Illn. 2021;17:257–68. 31483692 10.1177/1742395319872796

[6] Llanos C Gan EY Chen J Lee MJ Kilbreath SL Dylke ES. Reliability and validity of physical tools and measurement methods to quantify hand swelling: a systematic review. Phys Ther. 2021;101:pzaa206. 33313914 10.1093/ptj/pzaa206

[7] Beelen LM van Dishoeck AM Tsangaris E. Patient-reported outcome measures in lymphedema: a systematic review and COSMIN analysis. Ann Surg Oncol. 2021;28:1656–68. 33249519 10.1245/s10434-020-09346-0 PMC8693252

[8] Kikawa Y Uemura Y Taira T. A randomized study comparing electronic patient-reported outcome (ePRO) monitoring with routine follow-up during trastuzumab deruxtecan treatment in patients with metastatic breast cancer (PRO-DUCE study). J Clin Oncol. 2024;42:1504–1504.

[9] Wang AJ Hircock C Sferrazza D. The EORTC QLQ breast modules and the FACT-B for assessing quality of life in breast cancer patients: an updated literature review. Curr Opin Support Palliat Care. 2024;18:249–59. 39269251 10.1097/SPC.0000000000000724

[10] Demuro M Bratzu E Lorrai S Preti A. Quality of life in palliative care: a systematic meta-review of reviews and meta-analyses. Clin Pract Epidemiol Ment Health. 2024;20:e17450179183857. 39132583 10.2174/0117450179183857240226094258 PMC11311734

[11] Meilani E Zanudin A Mohd Nordin NA. Psychometric properties of quality of life questionnaires for patients with breast cancer-related lymphedema: a systematic review. Int J Environ Res Public Health. 2022;19:2519. 35270209 10.3390/ijerph19052519 PMC8909332

[12] Devoogdt N Van Kampen M Geraerts I Coremans T Christiaens MR. Lymphoedema functioning, disability and health questionnaire (Lymph-ICF): reliability and validity. Phys Ther. 2011;91:944–57. 21493748 10.2522/ptj.20100087

[13] Jamshidi F Farzad M MacDermid JC Varahra A Hosseini SA Asgarabad MH. Assessing the content based on ICF and quality based on COSMIN criteria of patient-reported outcome measures of functioning in breast cancer survivors: a systematic review. Breast Cancer. 2022;29:377–93. 35233732 10.1007/s12282-022-01340-6

[14] Paramanandam VS Lee MJ Kilbreath SL Dylke ES. Self-reported questionnaires for lymphoedema: a systematic review of measurement properties using COSMIN framework. Acta Oncol. 2021;60:379–91. 33475033 10.1080/0284186X.2020.1862422

[15] Zhao H Wu Y Tao Y. Psychometric validation of the Chinese version of the lymphedema functioning, disability, and health questionnaire for upper limb lymphedema in patients with breast cancer-related lymphedema. Cancer Nurs. 2022;45:70–82. 32541206 10.1097/NCC.0000000000000848

[16] Meilani E Zanudin A Nordin NAM. Psychometric evaluation of the Bahasa Malaysia version of the lymphedema functioning, disability, and health questionnaire for upper limb lymphedema in patients with breast cancer-related lymphedema. Lymphat Res Biol. 2024;22:210–20. 38608242 10.1089/lrb.2023.0045

[17] De Vrieze T Frippiat J Deltombe T. Cross-cultural validation of the French version of the lymphedema functioning, disability and health questionnaire for upper limb lymphedema (Lymph-ICF-UL). Disabil Rehabil. 2021;43:2797–804. 31990592 10.1080/09638288.2020.1716271

[18] Santos APO Rizzi SKLA Facina G Nazário ACP Elias S. Translation and cross-cultural adaptation of the LYMPH-ICF instrument for lymphedema into Portuguese/Brazil. Rev Bras Enferm. 2024;77:e20230137. 38896704 10.1590/0034-7167-2023-0137 PMC11178305

[19] Wild D Grove A Martin M; ISPOR Task Force for Translation and Cultural Adaptation. Principles of good practice for the translation and cultural adaptation process for patient-reported outcomes (PRO) Measures: report of the ISPOR task force for translation and cultural adaptation. Value Health. 2005;8:94–104. 15804318 10.1111/j.1524-4733.2005.04054.x

[20] Stefana A Damiani S Granziol U. Psychological, psychiatric, and behavioral sciences measurement scales: best practice guidelines for their development and validation. Front Psychol. 2025;15:1494261. 39916786 10.3389/fpsyg.2024.1494261 PMC11798685

[21] Terwee CB Mokkink LB Knol DL Ostelo RWJG Bouter LM de Vet HCW. Rating the methodological quality in systematic reviews of studies on measurement properties: a scoring system for the COSMIN checklist. Qual Life Res. 2012;21:651–7. 21732199 10.1007/s11136-011-9960-1 PMC3323819

[22] De Vrieze T Vos L Gebruers N. Revision of the lymphedema functioning, disability and health questionnaire for upper limb lymphedema (Lymph-ICF-UL): reliability and validity. Lymphat Res Biol. 2019;17:347–55. 30759059 10.1089/lrb.2018.0025

[23] Dos Santos Barros V Bassi-Dibai D Guedes CLR. Barthel index is a valid and reliable tool to measure the functional independence of cancer patients in palliative care. BMC Palliat Care. 2022;21:124. 35820921 10.1186/s12904-022-01017-z PMC9277778

[24] Mokkink LB Terwee CB Knol DL. The COSMIN checklist for evaluating the methodological quality of studies on measurement properties: a clarification of its content. BMC Med Res Methodol. 2010;10:22. 20298572 10.1186/1471-2288-10-22 PMC2848183

[25] Polit DF Beck CT. The content validity index: are you sure you know what’s being reported? Critique and recommendations. Res Nurs Health. 2006;29:489–97. 16977646 10.1002/nur.20147

[26] Cheung GW Cooper-Thomas HD Lau RS Wang LC. Reporting reliability, convergent and discriminant validity with structural equation modeling: a review and best-practice recommendations. Asia Pac J Manag. 2024;41:745–83.

[27] Cicchetti DV. Guidelines, criteria, and rules of thumb for evaluating normed and standardized assessment instruments in psychology. Psychol Assess. 1994;6:284–90.

[28] Peipert JD Cella D Hays RD. Significant individual change should be used as a lower bound for anchor based estimates of meaningful change on patient-reported outcome scores. Qual Life Res. 2024;33:3223–8. 39340723 10.1007/s11136-024-03788-9 PMC11599412

[29] Finch H. A comparison of methods for determining the number of factors to retain in exploratory factor analysis for categorical indicator variables. Psychol Int. 2025;7:3.

[30] Van Zyl LE ten Klooster PM. Exploratory structural equation modeling: practical guidelines and tutorial with a convenient online tool for Mplus. Front Psychiatry. 2022;12:795672. 35069293 10.3389/fpsyt.2021.795672 PMC8779472

[31] Khademi A Wells CS Oliveri ME Villalonga-Olives E. Examining appropriacy of CFI and TLI cutoff value in multiple-group CFA test of measurement invariance to enhance accuracy of test score interpretation. Sage Open. 2023;13:21582440231205354.

[32] Stone BM. The ethical use of fit indices in structural equation modeling: recommendations for psychologists. Front Psychol. 2021;12:783226. 34887821 10.3389/fpsyg.2021.783226 PMC8650002

[33] Schober P Boer C Schwarte LA. Correlation coefficients: appropriate use and interpretation. Anesth Analg. 2018;126:1763–8. 29481436 10.1213/ANE.0000000000002864

[34] Milkie MA Sayer LC Nomaguchi K Yan HX. Who’s doing the housework and childcare in America now? differential convergence in twenty-first-century gender gaps in home tasks. Socius. 2025;11:23780231251314667. 10.1177/23780231251314667 PMC12026444 40290637

[35] Sakuragi T Tanaka R Tsuji M; CORoNaWork Project. Gender differences in housework and childcare among Japanese workers during the COVID-19 pandemic. J Occup Health. 2022;64:e12339. 35781910 10.1002/1348-9585.12339 PMC9262311

[36] Moussa-Chamari I Farooq A Romdhani M. The relationship between quality of life, sleep quality, mental health, and physical activity in an international sample of college students: a structural equation modeling approach. Front Public Health. 2024;12:1397924. 39050600 10.3389/fpubh.2024.1397924 PMC11266085

[37] Byrne E Gaffey J Hayden L Daly A Gallagher P Dunne S. Body image and cancer-related lymphoedema: a systematic review. Psychooncology. 2023;32:1528–38. 37681525 10.1002/pon.6215

[38] Lau K Patel S Rogers K Smith S Riba M. Cancer-related lymphedema and psychological distress. Curr Psychiatry Rep. 2024;26:635–42. 39377989 10.1007/s11920-024-01543-y

[39] Hubel NJ Vorbach SM de Ligt KM. Sustainability and time trends in electronic patient-reported outcome assessment in routine cancer care: systematic scoping review and follow-up survey. J Med Internet Res. 2025;27:e69398. 40280556 10.2196/69398 PMC12064961

