# Supplementary material for: Psychometric validation of the Japanese version of the lymphedema functioning, disability, and health questionnaire for upper limb lymphedema: A multicenter cross-sectional study
Source: Medicine (Baltimore). 2026 Jul 24;105(30):e49846. doi: 10.1097/MD.0000000000049846 (PMC13406195; doi:10.1097/MD.0000000000049846)

**Supplemental Content 1.** Japanese version of the Lymphedema Functioning, Disability, and Health Questionnaire for Upper Limb Lymphedema.


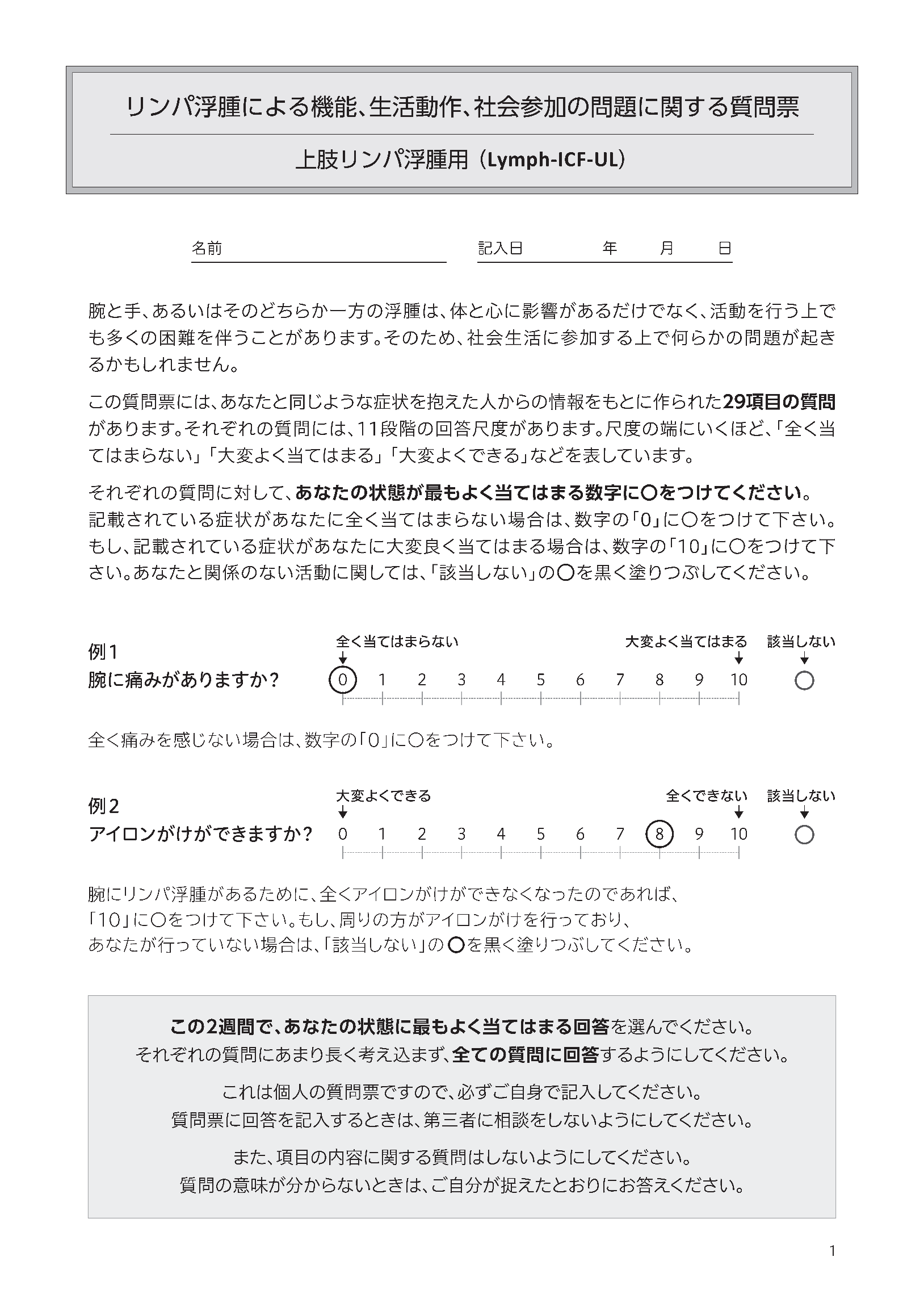


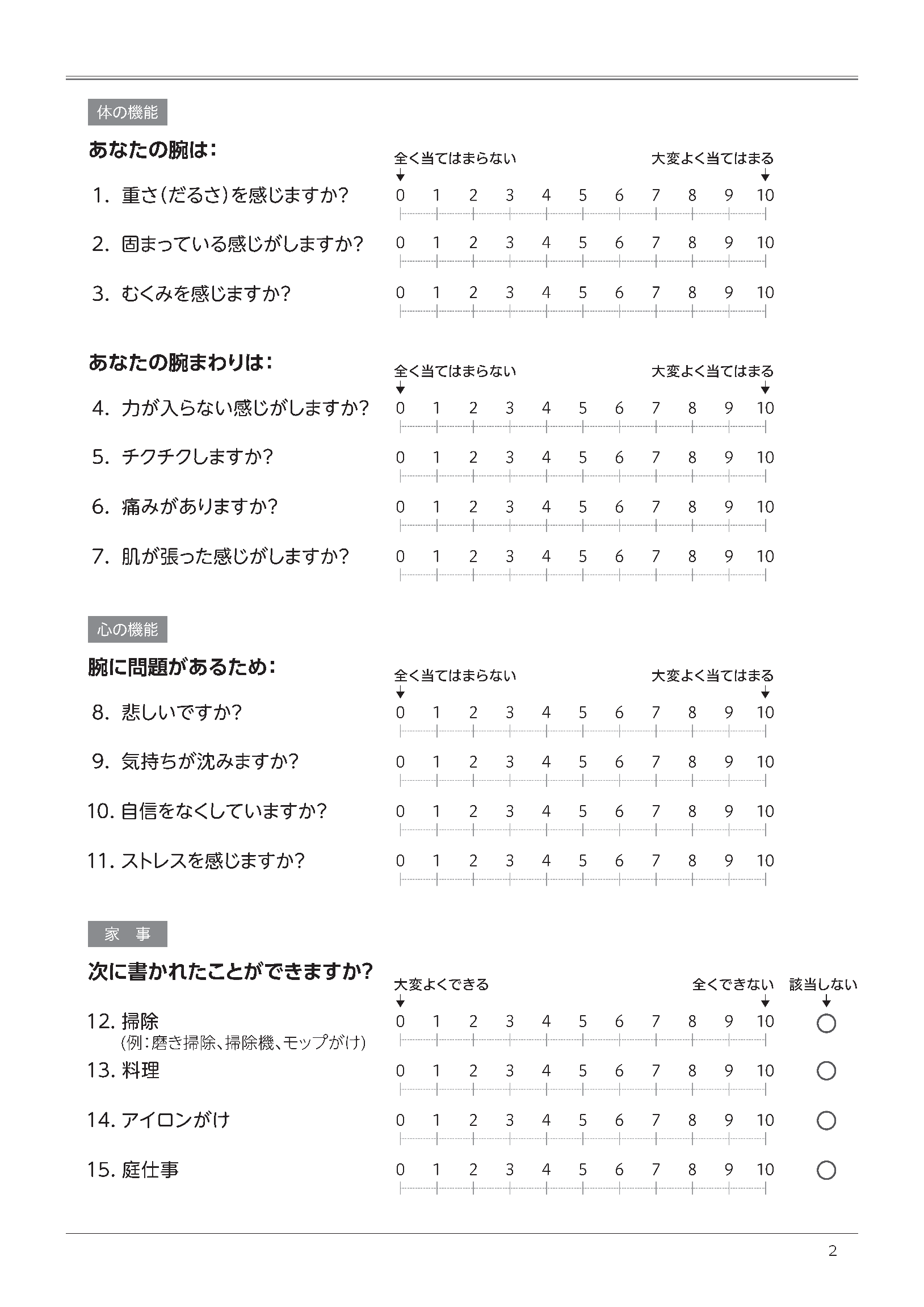


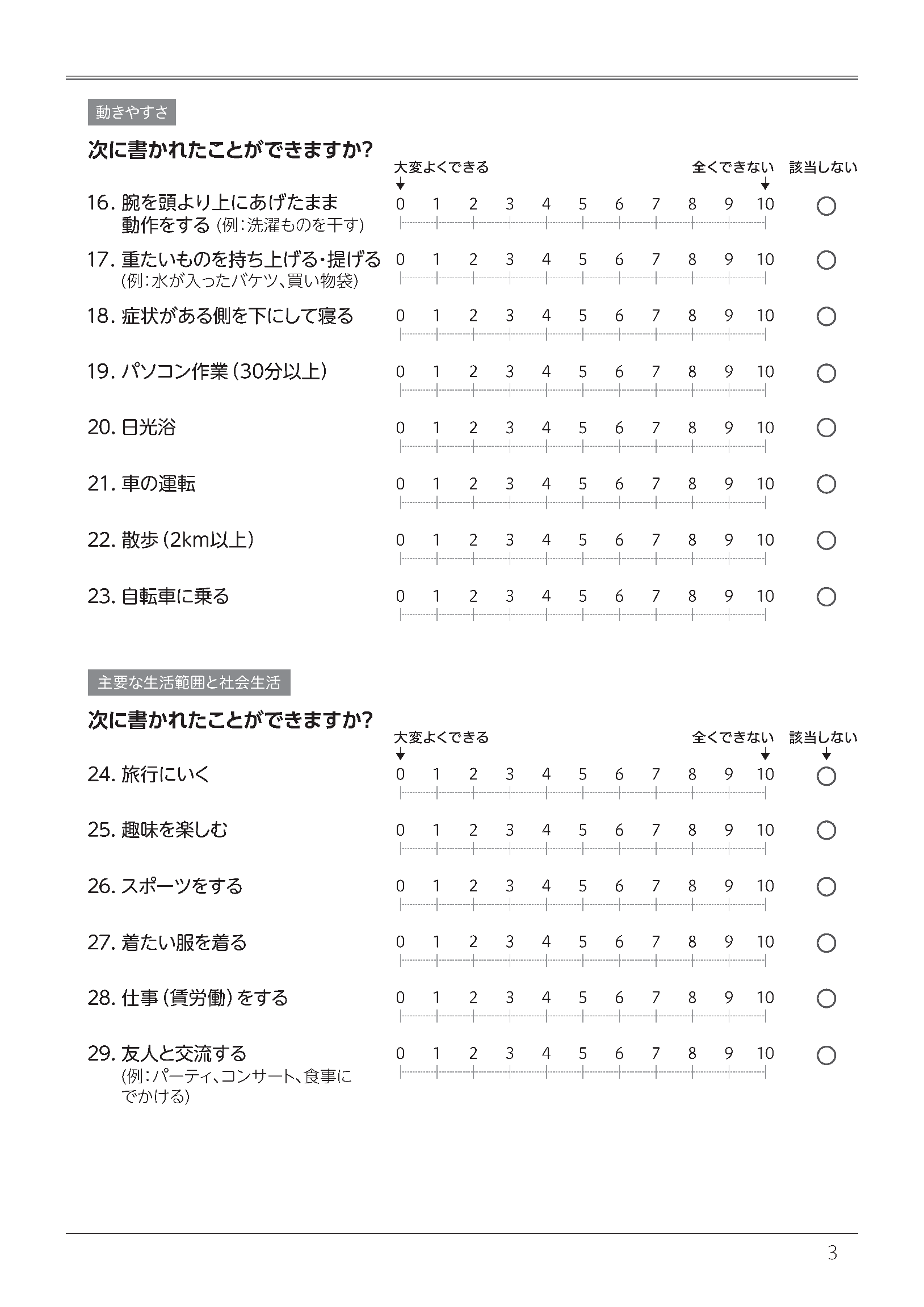


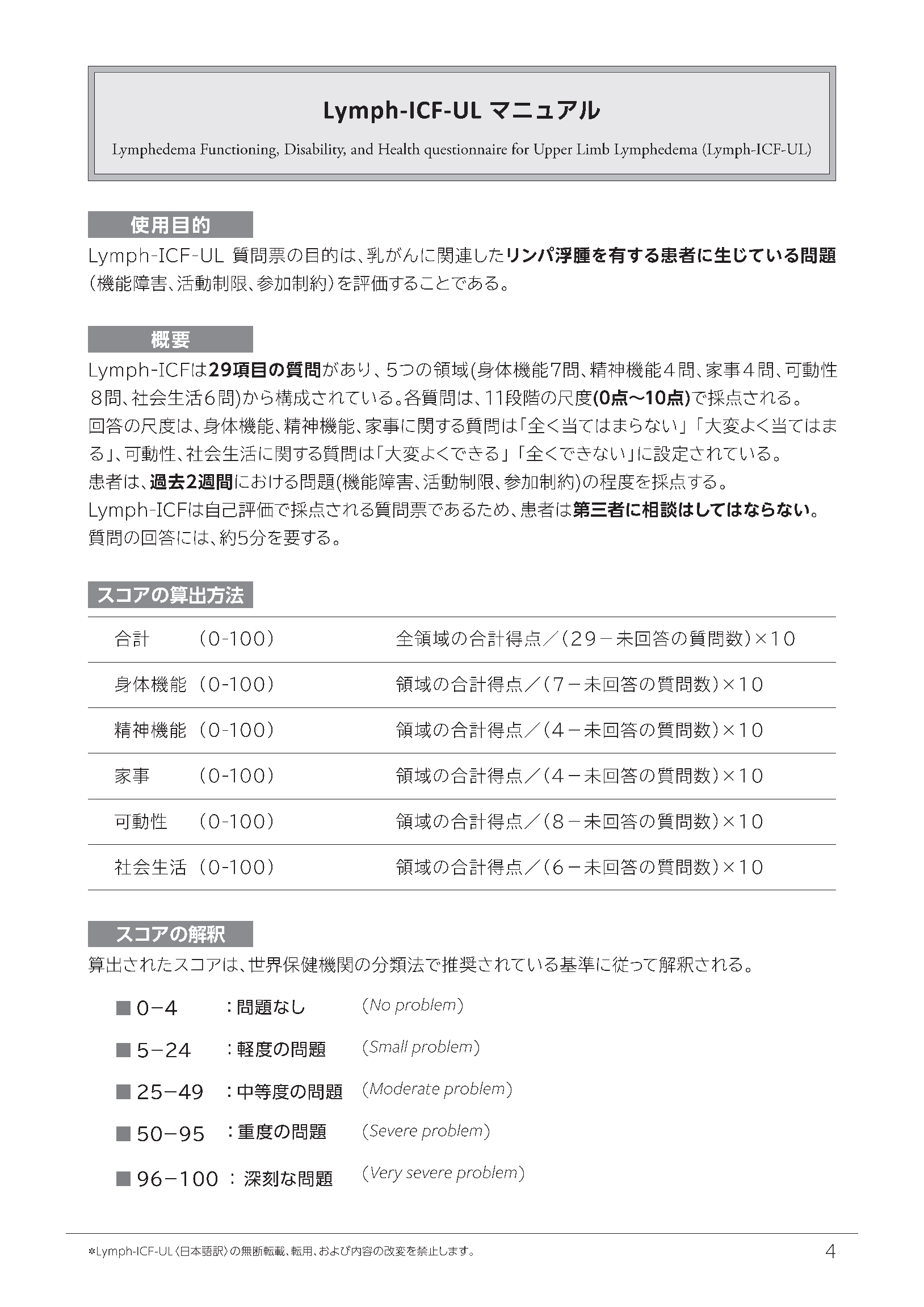

Supplement: Supplementary file 1 [file medi-105-e49846-s001.docx]
