# Supplementary material for: Psychometric validation of the Japanese version of the lymphedema functioning, disability, and health questionnaire for upper limb lymphedema: A multicenter cross-sectional study
Source: Medicine (Baltimore). 2026 Jul 24;105(30):e49846. doi: 10.1097/MD.0000000000049846 (PMC13406195; doi:10.1097/MD.0000000000049846)
Supplement: Supplementary file 2 [file medi-105-e49846-s002.docx]

**Supplemental Content 2.** Factor loadings for exploratory factor analysis.

| **Item　No.** | **Item Content (Abbreviated)** | **Exploratory-derived model** | | | |  | **Forced five-factor model** | | | | |
| --- | --- | --- | --- | --- | --- | --- | --- | --- | --- | --- | --- |
|  |  | **Factor 1** | **Factor 2** | **Factor 3** | **Factor 4** |  | **Factor 1** | **Factor 2** | **Factor 3** | **Factor 4** | **Factor 5** |
| Q1 | Heaviness | **0.903** | 0.096 | –0.163 | –0.028 |  | **0.896** | 0.113 | –0.048 | –0.089 | –0.046 |
| Q2 | Stiffness | **0.901** | –0.107 | –0.133 | 0.026 |  | **0.912** | 0.123 | –0.083 | –0.030 | 0.000 |
| Q3 | Swelling | **0.839** | –0.020 | –0.105 | 0.096 |  | **0.811** | –0.018 | 0.032 | –0.090 | 0.080 |
| Q4 | Weakness | **0.688** | 0.156 | –0.063 | 0.109 |  | **0.642** | 0.150 | 0.076 | –0.088 | 0.118 |
| Q5 | Tingling | **0.862** | –0.036 | –0.193 | 0.087 |  | **0.830** | –0.013 | –0.062 | –0.126 | 0.087 |
| Q6 | Pain | **0.853** | –0.095 | 0.364 | –0.320 |  | **0.801** | –0.068 | –0.249 | 0.217 | –0.337 |
| Q7 | Skin tightness | **0.912** | –0.194 | 0.018 | 0.026 |  | **0.899** | –0.166 | –0.016 | 0.048 | 0.009 |
| Q8 | Sadness | –0.013 | **0.895** | 0.205 | –0.162 |  | –0.014 | **0.857** | 0.178 | 0.143 | –0.170 |
| Q9 | Depression | 0.048 | **0.913** | 0.117 | –0.082 |  | 0.061 | **0.876** | 0.093 | 0.131 | –0.102 |
| Q10 | Low confidence | –0.014 | **0.889** | –0.010 | 0.076 |  | 0.345 | **0.876** | –0.113 | 0.145 | 0.050 |
| Q11 | Stress | 0.002 | 0.225 | –0.093 | –0.010 |  | 0.008 | 0.218 | –0.035 | –0.052 | –0.010 |
| Q12 | Housecleaning | 0.289 | 0.097 | **0.633** | –0.167 |  | 0.102 | 0.012 | **0.911** | –0.003 | –0.124 |
| Q13 | Cooking | 0.154 | 0.067 | **0.730** | –0.090 |  | –0.075 | –0.033 | **1.058** | –0.014 | –0.031 |
| Q14 | Ironing | 0.216 | –0.017 | **0.683** | –0.090 |  | 0.041 | –0.085 | **0.832** | 0.098 | –0.048 |
| Q15 | Gardening | 0.374 | 0.165 | **0.423** | –0.049 |  | 0.239 | 0.117 | **0.594** | 0.003 | –0.002 |
| Q16 | Overhead activity | 0.224 | –0.040 | 0.354 | **0.413** |  | 0.176 | –0.058 | 0.206 | 0.171 | **0.458** |
| Q17 | Lifting/carrying | 0.274 | 0.004 | 0.179 | **0.519** |  | 0.220 | –0.009 | 0.118 | 0.047 | **0.584** |
| Q18 | Sleeping on affected side | 0.257 | –0.052 | 0.166 | 0.363 |  | 0.240 | –0.046 | 0.017 | 0.131 | 0.389 |
| Q19 | Computer work | –0.123 | –0.092 | **1.100** | –0.062 |  | –0.112 | –0.075 | 0.320 | **0.842** | –0.072 |
| Q20 | Sunbathing | –0.223 | –0.075 | **0.833** | 0.062 |  | –0.094 | –0.017 | –0.155 | **0.934** | 0.005 |
| Q21 | Driving | –0.142 | –0.230 | **1.116** | –0.040 |  | –0.088 | –0.198 | 0.204 | **0.940** | –0.067 |
| Q22 | Walking | –0.106 | 0.086 | **0.664** | 0.098 |  | –0.011 | 0.124 | –0.097 | **0.738** | 0.059 |
| Q23 | Bicycling | 0.139 | 0.014 | **0.490** | 0.138 |  | 0.205 | 0.048 | –0.080 | **0.548** | 0.114 |
| Q24 | Traveling | 0.015 | –0.203 | 0.336 | 0.695 |  | –0.045 | –0.023 | 0.199 | 0.131 | **0.763** |
| Q25 | Hobbies | –0.022 | –0.006 | –0.239 | 1.060 |  | –0.025 | –0.025 | –0.230 | –0.123 | **1.116** |
| Q26 | Sports | 0.091 | 0.122 | 0.173 | **0.481** |  | 0.092 | 0.120 | –0.032 | 0.180 | **0.509** |
| Q27 | Clothing choice | 0.102 | **0.433** | 0.137 | 0.234 |  | –0.210 | 0.362 | **0.420** | –0.180 | 0.305 |
| Q28 | Paid work | –0.029 | 0.215 | **0.630** | –0.002 |  | 0.042 | 0.251 | –0.026 | **0.640** | –0.018 |
| Q29 | Socializing | –0.220 | –0.012 | **0.413** | **0.506** |  | –0.189 | –0.012 | –0.012 | 0.368 | **0.535** |
| Factor loadings were extracted using maximum likelihood estimation with Promax rotation. Factor loadings ≥ 0.40 are shown in bold. | | | | | | | | | | | |
