# Supplementary material for: Psychometric validation of the Japanese version of the lymphedema functioning, disability, and health questionnaire for upper limb lymphedema: A multicenter cross-sectional study
Source: Medicine (Baltimore). 2026 Jul 24;105(30):e49846. doi: 10.1097/MD.0000000000049846 (PMC13406195; doi:10.1097/MD.0000000000049846)
Supplement: Supplementary file 3 [file medi-105-e49846-s003.docx]

**Supplemental Content 3.** Convergent and discriminant validity.

|  | | **SF-36 subscale** | | | | | | | |
| --- | --- | --- | --- | --- | --- | --- | --- | --- | --- |
|  |  | **Physical functioning** | **Role-physical** | **Bodily pain** | **General health** | **Vitality** | **Social functioning** | **Role-emotional** | **Mental health** |
| Lymph-ICF-UL domain | Physical functions | –0.352 (<0.001) | –0.394 (<0.001) | **–0.514** † **(<0.001)** | –0.373 (<0.001) | –0.406 (<0.001) | –0.313 (<0.001) | **–0.371** † **(<0.001)** | **–0.332** † **(<0.001)** |
|  | Mental functions | **–0.278** † **(<0.001)** | **–0.318** † **(<0.001)** | –0.387 (<0.001) | –0.282 (<0.001) | –0.439 (<0.001) | –0.337 (<0.001) | –0.378 (<0.001) | **–0.476** † **(<0.001)** |
|  | Household activities | **–0.512** † **(<0.001)** | –0.539 (<0.001) | –0.452 (<0.001) | –0.326 (<0.001) | –0.489 (<0.001) | –0.453 (<0.001) | **–0.457 (<0.001)** | **–0.421 (<0.001)** |
|  | Mobility activities | **–0.638** † **(<0.001)** | –0.590 (<0.001) | –0.507 (<0.001) | –0.421 (<0.001) | –0.536 (<0.001) | –0.516 (<0.001) | **–0.486 (<0.001)** | **–0.418 (<0.001)** |
|  | Life and social activities | **–0.612 (<0.001)** | –0.522 (<0.001) | –0.490 (<0.001) | –0.388 (<0.001) | –0.526 (<0.001) | **–0.552** † **(<0.001)** | –0.452 (<0.001) | –0.431 (<0.001) |
| Values are Spearman’s rank correlation coefficients (*p*-value). A dagger (†) indicates that the correlation met the prespecified hypothesis. The hypotheses were derived from the original Lymph-ICF study and operationalized for the revised NRS-based Lymph-ICF-UL.^12,22^ Correlations in bold indicate those tested for construct validity hypotheses. The strength of the correlation was interpreted based on the absolute value of the correlation coefficient as follows: < 0.4 = weak, 0.4–0.74 = moderate, 0.75–0.9 = strong, and ≥ 0.9 = very strong. Convergent validity was supported when corresponding domains showed moderate-to-very strong correlations; discriminant validity was supported when the unrelated domains showed weak correlations.  Abbreviations: Lymph-ICF-UL, Lymphedema Functioning, Disability, and Health Questionnaire for Upper Limb Lymphedema; SF-36, 36-Item Short-Form Health Survey Questionnaire. | | | | | | | | | |
